# Characterizing the influence of skin pigmentation on pulse oximetry

**DOI:** 10.1117/1.BIOS.2.3.032506

**Published:** 2025-06-11

**Authors:** Arjun Putcha, Kevin Schichlein, Tien Nguyen, Brayden Davis, Rachel Choffin, Simran Malik, Aastha Sharma, Grace Sosa, Sara Saffari, Joseph Ribaudo, Jessica Guidici, Robert Lampman, John Eian, Michael Kosorok, Leonid Shmuylovich, Mitchell Pet, Wubin Bai

**Affiliations:** a University of North Carolina, Department of Applied Physical Sciences, Chapel Hill, North Carolina, United States; b University of North Carolina, Department of Toxicology & Environmental Medicine, Chapel Hill, Carolina, United States; c University of North Carolina/North Carolina State University, Joint Department of Biomedical Engineering, Chapel Hill, Carolina, United States; d University of North Carolina, Division of Clinical Laboratory Sciences, Chapel Hill, Carolina, United States; e University of North Carolina, Department of Health Policy & Management, Chapel Hill, Carolina, United States; f University of North Carolina, Department of Computer Science, Chapel Hill, North Carolina, United States; g Washington University, School of Medicine – Division of Plastic & Reconstructive Surgery, St. Louis, Missouri, United States; h University of North Carolina, School of Medicine – Division of Hospital Medicine, Chapel Hill, Carolina, United States; i University of North Carolina, Department of Biostatistics, Chapel Hill, Carolina, United States

**Keywords:** pulse oximetry, skin-tone bias, optoelectronics, photoplethysmography, hypoxemia

## Abstract

**Significance:**

Pulse oximeters are frequently used in healthcare settings to noninvasively estimate blood oxygen levels in patients, in turn influencing clinical decision-making. However, numerous studies have found pulse oximeters consistently overestimate oxygen levels in patients of color, with this overestimation increasing as oxygen levels decline. Although some reports postulate skin pigmentation may be the source of bias, to our knowledge, the precise impact of skin pigmentation on pulse oximetry has not been elucidated.

**Aim:**

Our objective is to experimentally characterize the impact of skin pigmentation on pulse oximeters across a broad range of oxygen saturations and determine whether skin pigmentation can explain the observed pulse oximeter bias in patients of color.

**Results:**

Using a live porcine model, we experimentally demonstrate the impact of skin pigmentation on pulse oximeter calculation. In particular, we show that skin pigmentation attenuates the sensitivity of red-light absorption to changes in oxygen saturation and show that the pulse oximeter output is consequently attenuated in pigmented skin.

**Conclusion:**

The systematic animal experiments confirm the confounding impact of skin pigmentation on intermediate calculations of pulse oximetry, and validate existing clinical observations of pulse oximeter bias in patients of color.

Statement of Discovery This work characterizes the impact of skin pigmentation via a porcine model on intermediate calculations and experimentally validates the correlation between skin-tone and pulse oximeter accuracy, consistent with existing clinical observations.

## 1 Introduction

Maintaining proper oxygen content of the blood is critical to the normal function of the body and its organs. For this reason, physicians rely on the accurate measurement of oxygen in the blood to make decisions, sometimes life-altering, about the amount of clinical support patients require. The most widely used measurement of blood oxygenation is blood oxygen saturation, which measures the percent of hemoglobin bound to oxygen.^1^^,^^2^

To measure the amount of oxygen that is being delivered to the body and its organs, physicians ideally look to direct measures of arterial blood oxygenation, including the partial pressure of oxygen (${\mathrm{PaO}}_{2}$) or the oxygen saturation of blood hemoglobin (${\mathrm{SaO}}_{2}$), both of which require an arterial blood gas (ABG). Unfortunately, ABGs have significant limitations as they require arterial needle puncture, an invasive procedure that, in contrast to the more common venipuncture, is often painful for the patient.^3^ ABGs also only provide information about the moment the sample was taken, i.e., they cannot provide continuous monitoring.^4^ These challenges led to the development of pulse oximeters. Pulse oximeters are optically based, noninvasive devices that continuously and indirectly estimate blood oxygen saturation (${\mathrm{SpO}}_{2}$) by measuring the light intensity ratio of typically two wavelengths of light absorbed by oxygenated hemoglobin and deoxygenated hemoglobin, as the light passes through or is reflected by the body.^1^^,^^2^ Pulse oximetry is now ubiquitous in surgical, postoperative, emergency medicine,^5^ inpatient hospitals, and outpatient ambulatory healthcare settings.^6^

Despite ABGs being the gold standard, pulse oximetry has largely supplanted arterial blood gases and is often the only measurement of oxygenation collected for patients. A 2018 emergency department study of patients presenting with chronic obstructive pulmonary disease (COPD), a common respiratory complaint, found that only 1.1% of patients had an ABG.^7^ In addition, a common emergency department nurse triage screening protocol, the “Emergency Severity Index Handbook,” requires a ${\mathrm{SpO}}_{2}$, rather than ${\mathrm{SaO}}_{2}$, for all presenting respiratory patients.^8^ Pulmonary emboli, which are a potentially life-threatening condition, are screened for by physicians utilizing the Pulmonary Embolism Severity Score and related Simplified Pulmonary Embolism Severity Score. These scoring tools were validated using ${\mathrm{SaO}}_{2}$,^9^^,^^10^ but commonly used online calculator versions permit the use of ${\mathrm{SpO}}_{2}$ instead. Medicare has specified that an arterial blood saturation of less than 88% is required to determine whether a patient beneficiary qualifies for home oxygen. As of 2023, Medicare explicitly allows either blood gases or pulse oximetry to be used for oxygen saturation, further establishing the ubiquity of pulse oximeters in healthcare.^11^

Despite the ubiquity and ease of use, there are growing concerns about decreased accuracy of ${\mathrm{SpO}}_{2}$ compared with ${\mathrm{SaO}}_{2}$, particularly in racial or ethnic minorities. Studies across multiple disease processes and hospital settings have consistently found that ${\mathrm{SpO}}_{2}$ overestimates oxygenation in patients of color (most commonly in Black patients) compared with the gold standard ${\mathrm{SaO}}_{2}$ values.^12^^–^^18^ Consistent with these studies, Valbuena et al. found that pulse oximeters failed to detect hypoxemia 21.5% of the time in black patients—more than twice as frequently as any other race in the study. This phenomenon is termed occult hypoxemia.^19^ Given the importance of ${\mathrm{SpO}}_{2}$ measurements, where single-digit valuation changes directly impact patients, these studies indicate that racial and ethnic minorities may be receiving disproportionately inadequate treatment based on the false reassurance of their ${\mathrm{SpO}}_{2}$ readings.

Within the medical community, it is well-known that there are elevated rates of ICU mortality in racial minorities.^20^ It is possible that a technological gap, namely the inaccuracy of pulse oximeter data for racial and ethnic minorities, may be contributing to this mortality gap. It is generally understood that pulse oximetry can be affected by many confounding factors, including low perfusion, motion, temperature, skin pigmentation, ambient light, and others.^21^ In addition, several researchers have identified mechanisms to improve accuracy of pulse oximetry under the aforementioned conditions by modifying the light intensity emitted from the pulse oximeter,^22^ changing the pulse width of light flashes,^23^ using radially polarized light to measure oxygenation,^24^ changing the color of the light used to obtain oxygenation status,^25^ and changing the spectral width of light emission.^26^ Although the above methodologies can improve pulse oximeter accuracy in various conditions, and some appear to be particularly accurate in patients of color, to our knowledge, no mechanism has been found that performs systematically in patients regardless of skin tone across oxygen saturation levels. This may be due to insufficient availability of evidence identifying a specific source of the observed bias in pulse oximetry, thus preventing targeted methodologies from being developed.

In the following work, we present a mathematical framework to describe pulse oximeter data from a time and frequency domain perspective. Leveraging the naturally occurring spotted pigmentation present in Hampshire swine, we then use this split-pigmentation porcine model to assess the impact of skin pigmentation on pulse oximetry across a broad range of oxygen saturations. In particular, we rely on our mathematical framework to characterize the impact of skin pigmentation on the absorbance of red and near-infrared light, and in turn, their impact on pulse oximetry. These experiments demonstrate a statistically significant impaired sensitivity of red light to changes in oxygen saturation in pigmented skin. Together, this work validates existing clinical observations of pulse oximeter bias in patients of color and characterizes the impact of skin pigmentation on intermediate pulse oximeter calculations.

### 1.1 Photoplethysmography and Pulse Oximetry

**Fig. 1:**
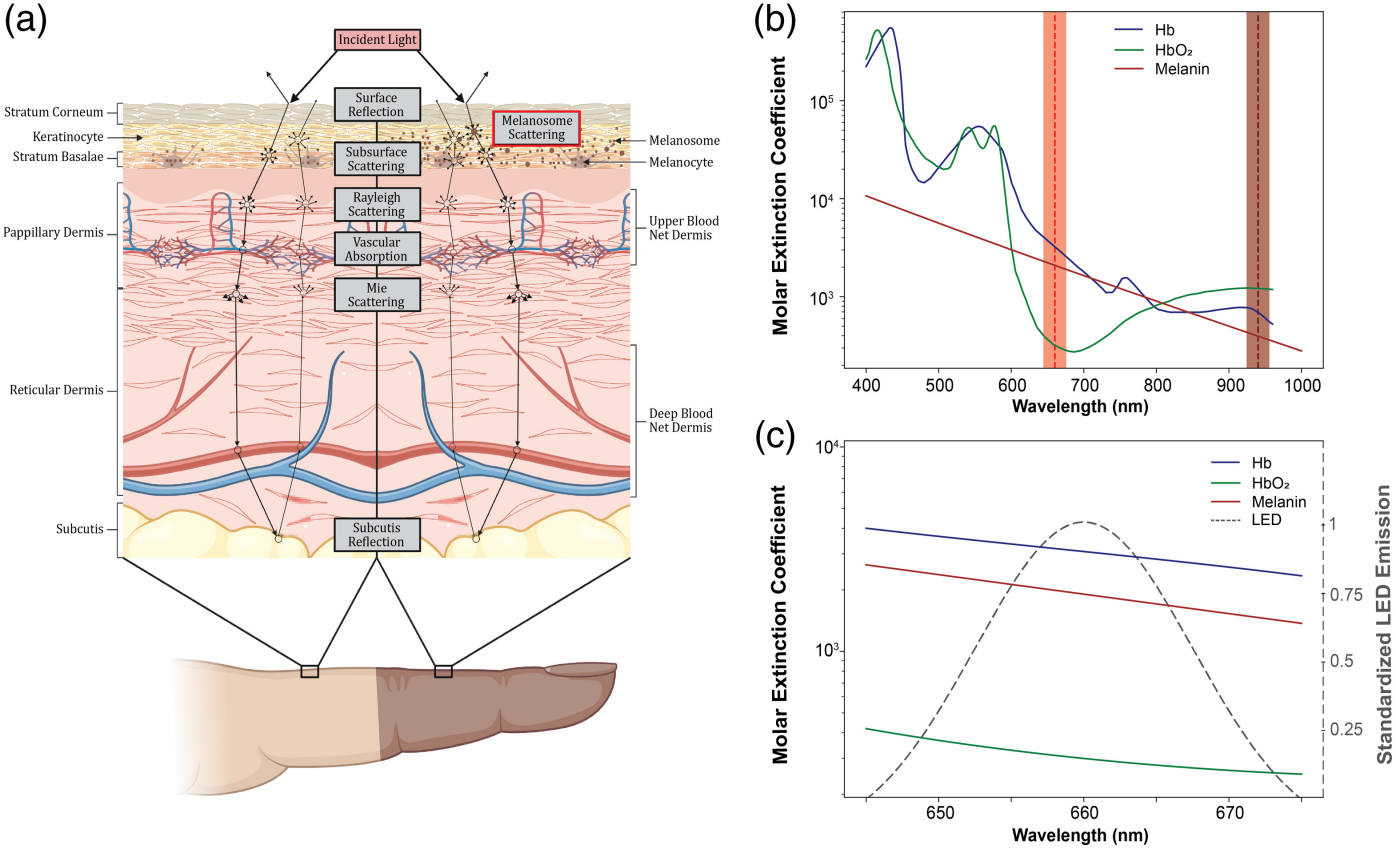
(a) Tissue morphology and light–tissue interactions. (b) Absorption coefficients of oxyhemo globin (${\mathrm{HbO}}_{2}$), deoxyhemoglobin (Hb), and melanin from 400 to 1000 nm. Highlighted regions represent lightsource spectral emission for pulse oximetry. (c) Standardized absorption of red-LED light through a medium of hemoglobin and melanin. Molar Extinction Coeffecient curves come from Scott Prahl’s experimental work.^45^

Because noninvasive pulse oximetry is often assessed on the fingertip, we first consider the morphology of structures within tissue we expect to find in the index finger, as shown in Fig. 1(a). Light must travel sequentially through each layer of tissue, interacting with various chromophores, before being measured by a photodiode. Because we are focused on how skin pigmentation affects pulse oximetry, we center our discussion on two relevant features: skin pigmentation and blood oxygenation. Skin pigmentation arises from chromophores in the epidermis; specifically, melanocytes produce melanosomes—organelles that can distribute within the epidermis—that in turn produce melanin, the chromophore responsible for pigmentation. Although melanocyte concentration does not change between skin pigmentations,^27^^–^^29^ in Fig. 1(a), it is apparent that melanosome size and number are higher in pigmented skin, and melanin production is also increased.^30^^–^^32^

Once light passes the epidermis, a proportion of the remaining light will be absorbed by hemoglobin within the blood in the capillary networks in the dermis.^33^ The illuminated blood, especially in the upper capillary network, dictates the assessment of pulse oxygenation. It is therefore sufficient to note that the majority of the red light is absorbed within the epidermis, whereas a significant portion of infrared light is absorbed by both the epidermis and dermis.^34^^,^^35^ Although there are many other chromophores to consider, for the purposes of our discussion on skin pigmentation, those described above provide a sufficiently simplified model of light–tissue interactions in the context of pulse oximetry.

The mechanism through which we estimate ${\mathrm{SpO}}_{2}$ is through *photoplethysmography* (PPG)—a technique that measures changes in the blood volume of vascularized tissue. More specifically, optical radiation is used to illuminate tissue structures, where it is scattered, absorbed, and reflected in various proportions as it passes through various media before being transmitted through the tissue or reflected back to the tissue surface. For further discussion on optical physics, see Supplementary Material, Note 2. This attenuated light is detected by a photodetector and recorded as a voltage signal over time, thus creating a PPG signal.^36^^,^^37^ By measuring changes in light transmission or reflectance over time, PPGs can reflect variations in the attenuation of light due to changes in tissue composition, such as oxygen status and cardiac activity.^38^^–^^40^

Because hemoglobin holds 97–98% of the total oxygen content within blood, the measurement of concentration ratios between Hb and ${\mathrm{HbO}}_{2}$ provides a good approximation of blood oxygen levels. As shown in Fig. 1(b), because Hb and ${\mathrm{HbO}}_{2}$ differentially absorb red and near-infrared (IR) light—typically, 660 and 940 nm wavelengths, respectively—the relative absorbance of these two wavelengths can be used to estimate the blood oxygen level, ${\mathrm{SpO}}_{2}$. To be more precise, by taking the ratio of absorbance of each wavelength, we indirectly estimate the proportion of hemoglobin that is bound to oxygen.

Pulse oximeters generally make use of light-emitting diodes (LEDs) and photodiodes (PDs) to emit and measure red and NIR light. The photodiode measures the transmission or reflectance of light passing through the tissue—a proxy for attenuation. Changes in attenuation over time can then be interpreted to be a function of changes in blood flow (a function of cardiac output) and blood oxygenation (${\mathrm{SpO}}_{2}$). To improve the inter-patient interpretability of the PPG signal, the measurement of the pulsatile signal (resulting from heartbeats) is often divided by the baseline light measurement. This process is done for both red and NIR data, and the ratio of the two yields the *modulation ratio* (R), which is then mapped to an experimentally derived curve to estimate blood oxygen levels. This is often written as

$$R=\frac{dPA_{\text{overall}}^{660\;\mathrm{nm}}}{dPA_{\text{overall}}^{940\;\mathrm{nm}}}\approx \frac{{\mathrm{PA}}_{\mathrm{AC}}^{660\;\mathrm{nm}}/{\mathrm{PA}}_{\mathrm{DC}}^{660\;\mathrm{nm}}}{PA_{\mathrm{AC}}^{940\;\mathrm{nm}}/{\mathrm{PA}}_{\mathrm{DC}}^{940\;\mathrm{nm}}}=\frac{{\mathrm{PA}}_{\mathrm{AC}}^{660\;\mathrm{nm}}\cdot {\mathrm{PA}}_{\mathrm{DC}}^{940\;\mathrm{nm}}}{{\mathrm{PA}}_{\mathrm{AC}}^{940\;\mathrm{nm}}\cdot {\mathrm{PA}}_{\mathrm{DC}}^{660\;\mathrm{nm}}}=R_{\mathrm{AC}}\cdot R_{\mathrm{DC},}$$

where ${\mathrm{PA}}_{\mathrm{AC}}^{\lambda }$ refers to the pulsatile photodiode absorption of light, ${\mathrm{PA}}_{\mathrm{DC}}^{\lambda }$ is the baseline photodiode absorption of light at wavelength λ, $R_{\mathrm{AC}}$ refers to the ratio of pulsatile absorption, and $R_{\mathrm{DC}}$ is the baseline ratio. For a further discussion of pulse oximeter instrumentation, see Supplementary Material, Note 3. It is critical to note that signal recordings from a photodiode are *not* the direct measurement of the absorption of light by tissue; rather, photodiode measurements are modulated by the collective attenuation of light interacting with tissue—determined by absorption, reflection, and scattering interactions.

### 1.2 PPG Formulation

Melanin’s absorption spectrum has the unique property of an exponential dependence on wavelength;^41^^,^^42^ The consequence of this feature is that the absorption of red light by the epidermis will be substantially higher than that of infrared light. As shown in Fig. 1(b), due to this exponential dependence, the change in absorption due to melanin within the red region of light (640 to 680 nm) is also greater than that of infrared light (920 to 960). Because LEDs in practice generate a spectrum of wavelengths centered around a peak wavelength, as shown in Fig. 1(c), the measured absorption of light is based on a convolution of relative LED spectral emission over the absorption of tissue components—including hemoglobin and melanin. As shown in Fig. 4(a), light propagates sequentially through each layer of the skin, with the epidermis interacting with light before the dermis—where the physiologically relevant signal occurs. Therefore, the measured photodiode signal can be represented as

$$\mathrm{PPG}(\mathrm{LED},t)=\int_{\lambda_{1}}^{\lambda_{2}}\Lambda^{\mathrm{PD}}(\lambda )[\Lambda_{\mathrm{tot}}^{\mathrm{LED}}(t)-\int_{\lambda_{1}}^{\lambda_{2}}\Lambda^{\mathrm{LED}}(\lambda )\overset{M}{\underset{m=1}{\sum }}\rho_{m}^{\lambda }[X(t){]}_{m}\varepsilon_{m}^{\lambda }l_{m}^{\lambda }(t)d\lambda ]d\lambda ,$$

where $\lambda_{1}$ and $\lambda_{2}$ are the bounds for the LED spectral emission (generally wider for longer wavelength LEDs), $\Lambda_{\mathrm{tot}}^{\mathrm{LED}}$ is the total LED emission, $\Lambda^{\mathrm{LED}}(\lambda )$ is the relative LED spectral emission at wavelength λ, $\Lambda^{\mathrm{PD}}(\lambda )$ is the relative current response of the photodiode to a given wavelength of light, M is the number of distinct chromophores in the tissue, t is the timepoint, and $[X]\varepsilon^{\lambda }l^{\lambda }$ is Beer-Lambert’s law for a given chromophore X and wavelength λ, and $\rho_{i}^{\lambda }$, the *attenuation coefficient*, is the proportion of light attenuation for a given wavelength λ and chromophore i. From this, the baseline (DC) and pulsatile (AC) components of photodiode absorption at a fixed point in time can be written as

$$R_{\mathrm{DC}}=\frac{{\mathrm{PA}}_{\mathrm{DC}}^{940\;\mathrm{nm}}}{{\mathrm{PA}}_{\mathrm{DC}}^{660\;\mathrm{nm}}}=\frac{\int_{t1}^{t2}\mathrm{PPG}(\mathrm{IR},t)dt}{\int_{t1}^{t2}\mathrm{PPG}(\mathrm{RED},t)dt},$$

$$R_{\mathrm{AC}}=\frac{{\mathrm{PA}}_{\mathrm{AC}}^{660\;\mathrm{nm}}}{{\mathrm{PA}}_{\mathrm{AC}}^{940\;\mathrm{nm}}}=\frac{\max(\mathrm{PPG}(\text{Red},\overset{\to }{t}))-\min(\mathrm{PPG}(\text{Red},\overset{\to }{t}))}{\max(\mathrm{PPG}(\mathrm{IR},\overset{\to }{t}))-\min(\mathrm{PPG}(\mathrm{IR},\overset{\to }{t}))},$$

where t1 and t2 are the start and end of an analysis window, $\vec{t}$ is the vector of all timepoints contained within, and *Red* refers to an LED with an approximately Gaussian spectral emission curve centered at 660 nm, whereas IR represents the same centered at 940 nm. It should be noted that we make an implicit assumption that each chromophore i receives the same proportion of photons regardless of x-y location, and a given chromophore must be approximated to the same depth. Although this is an imprecise definition, expanding the chromophore list to separate the same chromophore at different depths would expand the applicability of this model to human tissue. Therefore, $\rho_{i}^{\lambda }$ is closer to 1 at layers of tissue near the surface, and the value of $\rho_{i}^{\lambda }$ for a layer 2 chromophore is directly dependent on the attenuation accomplished by layer 1.

In addition, because $\rho_{i}^{\lambda }$ represents light attenuation, it encompasses the impacts of absorption in addition to scattering and reflection. When considering the impact of light scattering due to skin pigmentation, Zonios et al. find that the scattering coefficient and gamma are less correlated in melanocytic nevi, suggesting other factors, such as melanosomes, could play a role in changes in scattering intensity. In addition, Song et al. predicted an exponential dependence on the scattering coefficient across 400 to 1000 nm, suggesting a greater degree of light-scattering in red wavelengths than in NIR.^43^ Finally, Tsang et al. found that melanosome size correlates with the extent of light scattering.^44^ Combined with our understanding of pigmented skin morphology, the above suggests pigmented skin should undergo a greater degree of light scattering, in addition to more light absorption, particularly in the red region of light, thereby increasing light attenuation by the epidermis.

From this, we can represent the PPG signal in the frequency domain using the discrete Fourier transform (DFT), as follows:

$${\mathrm{PPG}}_{k}=\overset{T-1}{\underset{t=0}{\sum }}\mathrm{PPG}(t)e^{-\frac{2\pi ikt}{T}}\;=\overset{T-1}{\underset{t=0}{\sum }}[\int_{\lambda_{1}}^{\lambda_{2}}\Lambda^{\mathrm{PD}}(\lambda )[\Lambda_{\mathrm{tot}}^{\mathrm{LED}}(t)-\int_{\lambda_{1}}^{\lambda_{2}}\Lambda^{\mathrm{LED}}(\lambda )\overset{M}{\underset{m=1}{\sum }}\rho_{m}^{\lambda }[X(t){]}_{m}\varepsilon_{m}^{\lambda }l_{m}^{\lambda }(t)d\lambda ]d\lambda ]e^{-\frac{2\pi ikt}{T}}\;\text{for}\;0\leq k\leq T-1.$$

For further discussion on the DFT, see Supplementary Material, Note 4. Looking at Fig. 4(a), we simulated the standardized spectral emission of red light (660 nm) as it interacts with each layer of tissue as described in Section 2.1. Here, it is apparent that pigmentation markedly attenuates the light available to interact with the succeeding layer—the vasculature—in addition to the light measured at the photodiode. Given that melanin and melanosomes have more light interactions in shorter wavelengths, it is reasonable to assume light is particularly attenuated in the red region when compared to the infrared. Given that deoxyhemoglobin absorbs red light more than infrared, it is possible that the attenuation of the red-region light by skin tone could explain why pulse oximeters perform worse at lower oxygen saturations, where deoxyhemoglobin dominates vascular absorption. Therefore, the mathematics framework describes a confounding effect from tissue chromophores (e.g. skin melanin and melanosomes) other than hemoglobin in modulating PPG recordings and the associated calculations of modulation ratio and blood oxygen levels, with the following experimental observations from a live porcine model providing further insights.

**Fig. 2:**
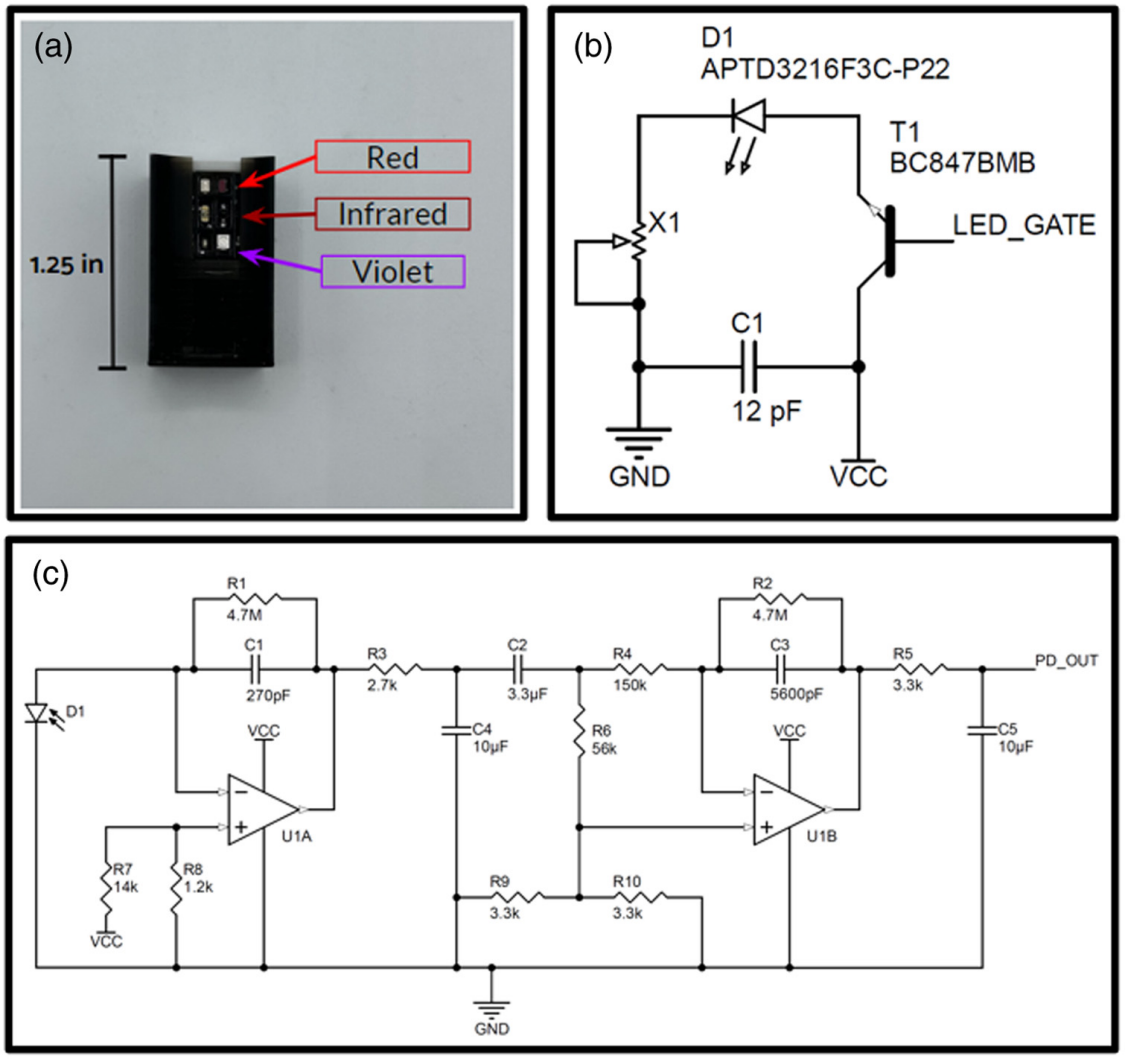
(a) Image of pulse oximeter used for porcine experiments. (b) LED driver circuit. X1 is a variable resistor, D1 is an LED, T1 a BJT transistor, and the power supply terminals are GND and VCC. (c) Photodiode signal conditioning circuit.

## 2 Methods

### 2.1 Simulation

Because our focus is on how skin pigmentation affects pulse oximetry, we chose to focus on the spectral characteristics of light interacting with four key chromophores in skin: melanin, melanosomes, deoxyhemoglobin, and oxyhemoglobin. Because the first two chromophores are responsible for skin pigmentation differences, and the latter two account for changes in oxygen saturation, considering these four chromophores provides a minimal basis to understand how skin pigmentation interacts with oxygen saturation in pulse oximetry.

To determine the spectral emission profile of light passing through each “layer” of the tissue, we first model LED spectral emission as a gaussian probability distribution centered at the peak wavelength of emission—660 nm for red, 940 nm for infrared—and a full-width half-maximum (FWHM) of 30 nm for both LEDs, a common value for surface-mount LEDs of both wavelengths. When considering the interaction with chromophores—where we use absorptivity coefficients derived from Scott Prahl’s work^45^—we find the maximum cumulative absorbance of all chromophores together and define the total light emission—in terms of number of photons—to be 10 times larger. We then define each condition as a fixed combination of absorbances. Melanin is 78% eumelanin and 22% pheomelanin, where one unit of melanin corresponds to light pigmentation, and five units to dark pigmentation—reflecting the reasonable range of melanin concentration in the human skin. Similarly, ${\mathrm{SaO}}_{2}$ is defined by the proportion of oxyhemoglobin and deoxyhemoglobin. Regardless of the skin tone, we keep the depth of the epidermis to 1 mm, and the dermis to 3 mm. Finally, for each condition—pigmentation level and ${\mathrm{SaO}}_{2}$—we calculate the cumulative absorption for each wavelength, convolve it with the LED emission curve, and subtract it from the total emission for each wavelength.

Finally, we also standardize the output transmission to the maximum value from the LED across wavelengths to make a reasonable comparison. In addition, we perform the above when considering solely the LED emission, the LED emission with melanin and melanosome presence, and finally, the LED emission with melanin, melanosome, and hemoglobin presence at 80% ${\mathrm{SaO}}_{2}$.

### 2.2 Device Design

Figure 2(a) depicts the reflectance-mode pulse oximeter used for the porcine experiments. Although we have three LED photodiodes (red, NIR, and violet) depicted, for the purposes of this work, we made use of only the red and NIR. Figure 2(b) depicts the LED driver circuit used to drive each LED while allowing for tunable brightness through two mechanisms: (1) pulse width modulation through the BJT transistor gate and (2) variable resistance using a digital potentiometer. Figure 2(c) depicts the photodiode signal conditioning circuit used in the analog signal processing pipeline to reduce noise prior to digital signal processing. Here, we have two primary stages of conditioning: (1) an active low-pass filter (125 Hz cutoff) using a transimpedance amplifier following by passive low-pass (7 Hz), notch (60 Hz), and high-pass filters (0.8 Hz), and (2) an active low-pass filter (6 Hz) followed by a passive low-pass filter (5 Hz) that feeds into an analog-to-digital converter (ADC) on an nRF 52840 ItsyBitsy Development Board uniformly sampling at 120 Hz, where every three samples are averaged to minimize the influence of noise—resulting in a functional sampling frequency of 40 Hz. This development board is then connected to a separate laptop to handle further processing.

### 2.3 Data Acquisition

To handle real-time data acquisition and calibration of devices during the experiment, we made use of Fastplotlib—a fast plotting library using the PyGFX rendering engine—to plot data in real-time.^46^ In addition, we made use of multiprocessing to handle the simultaneous acquisition of serial data, visualization of the data, and storage of the data into a SQLite file. More specifically, we initialize a shared-memory object to continuously acquire and store sensor data in a local buffer, and rely on mutexes to coordinate access to this buffer by plotting and storage processes.

### 2.4 Digital Signal Processing

**Fig. 3:**
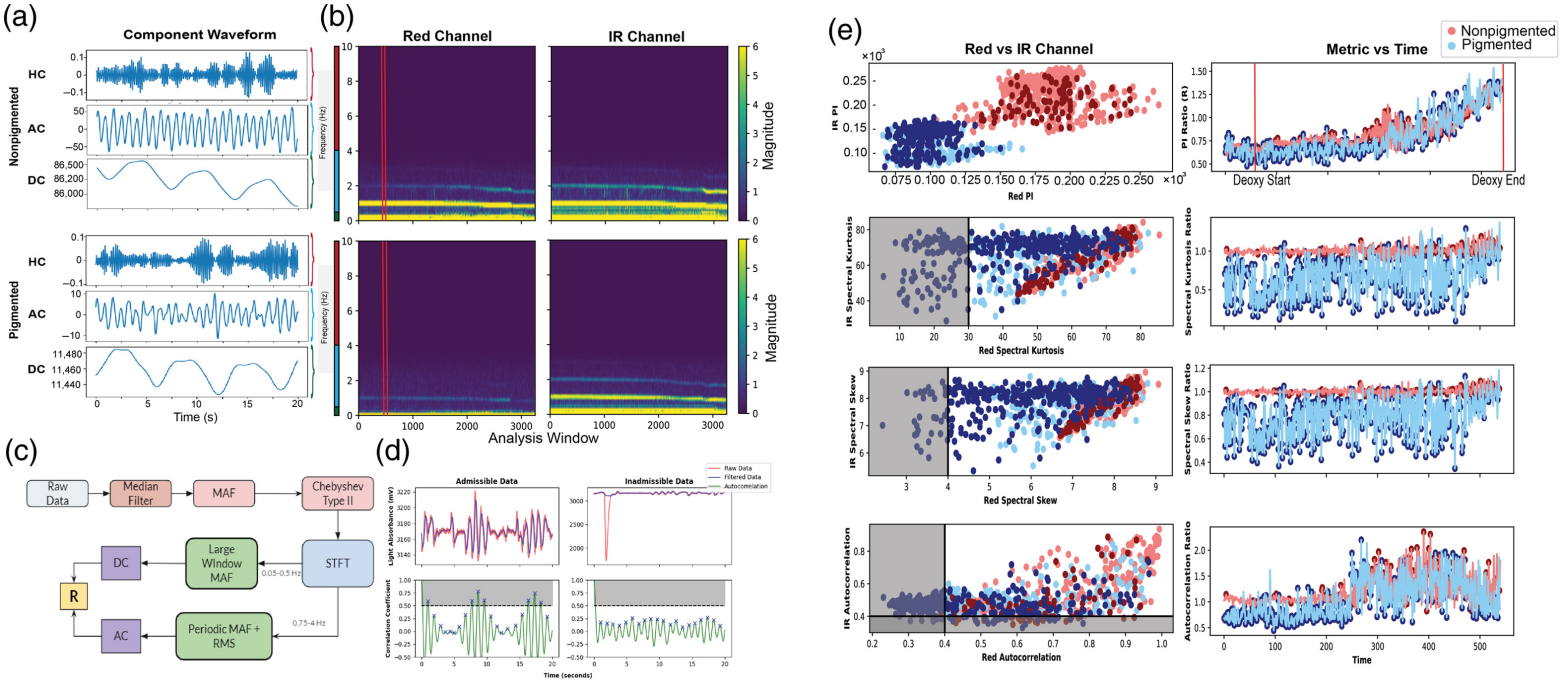
(a) Extracted pulsatile (AC), baseline (DC), and high-frequency (HC) components. (b) Frequency domain representation of red and infrared PPG data in pigmented and nonpigmented skin. (c) Digital signal processing pipeline. (d) Example of filtering data using our DSP pipeline and autocorrelation validation metric. (e) Validation metrics—PI, spectral entropy, spectral kurtosis, and autocorrelation—for red and infrared PPG data on pigmented and nonpigmented skin. Gray-shaded regions represent values that fail validation metrics.

Figure 3(c) shows the steps we took to filter our raw data acquired from the device. To attenuate the influence of high-intensity noise, we applied a median filter with a window size of 10 samples to the raw input data. Following this, we clipped points above and below a given global threshold—values more than 10% from the mean signal value of the entire dataset—and linearly interpolated these clipped values. Then, we applied an exponentially weighted moving average filter (EW-MAF) to further reduce high-frequency noise. We then applied a fourth-order Chebyshev Type II Low Pass Filter, as Liang et al. suggested this filter may be more effective in improving PPG signal quality.^47^

To extract the pulsatile (AC) component, we applied a fast Fourier transform (FFT), removed frequencies outside of the bandpass region (0.75 to 4 Hz), and applied an inverse FFT (iFFT). We then applied a periodic moving average filter as proposed by Lee et al. to attenuate motion artifacts between pulses, leveraging the quasi-periodicity of PPG waveforms.^48^ Finally, we took the root mean square of the signal, thereby giving the AC component magnitude.

To extract the baseline (DC) component of the PPG, we applied an FFT and extracted the frequencies below 0.5 Hz, applied an iFFT, and then applied a large-window moving average filter across the resulting signal. The resulting waveform proved to be an adequate representation of the DC component of absorption. Because we repeat this analysis across multiple time windows, we are in effect computing a short-time Fourier transform (STFT).

To extract the high-frequency (HC) component, we applied an FFT and removed frequencies below 6 Hz and then performed an iFFT. The resulting waveform contains the information for the high-frequency information in our PPG.

Figure 3(b) shows the frequency domain of the acquired data across time. Figure 3(a) shows the extracted AC, DC, and high-frequency (HC) components extracted from the frequency domain at a given timepoint by applying the bandpass filters mentioned above. Although both AC and DC components exhibit quasi-periodic motion, the frequency of occurrence in the AC component is due to the heartbeat, and the DC component is likely due to respiration patterns, which generally have a frequency between 0.25 and 0.75 Hz. Figures 3(c) and 3(d) show the impact of our filtering pipeline on admissible and inadmissible samples of data. Here, it is important to note that the determination of ‘inadmissibility’ is a function of validation metrics – discussed in the following section—not the filtering pipeline itself. In Fig. 3(d), we depict utilizing the autocorrelation metric as an indicator of data validity and demonstrate its utility in continuous assessment of data validity across time.

### 2.5 Signal Quality Indices

We use Signal Quality Indices (SQIs) to select reasonably interpretable analysis windows; specifically, we set thresholds for each metric that an analysis window must pass to be usable for subsequent analysis. In doing so, we avoid drawing conclusions from low-quality data, thus improving the generalizability of our analysis.

***Pulsatility Index (PI)***: The pulsatility index is the ratio of the pulsatile blood flow to the non-pulsatile or static blood in peripheral tissue. In other words, it is the difference in the amount of light absorbed through the pulse when light is transmitted through the finger, which can be defined as follows:

$$\mathrm{PI}=\frac{x_{\max}-x_{\min}}{\mu_{x}}\approx \frac{\mathrm{AC}}{\mathrm{DC}},$$

where PI is the pulsatility index and $\mu_{x}$ is the statistical mean of the x signal (filtered PPG signal). PI can therefore be roughly approximated by the AC component divided by the DC component, where each component can be extracted through time-domain or frequency-domain techniques.

***Relative power spectral density (rPSD)***: The frequency domain was explored to assess the PPG signal quality, a different perspective from the time domain feature discussed above. Because most of the energy of the systolic and diastolic waves (culminating in the observed heart beat) is concentrated within the 0.75 to 4 Hz frequency band, the ratio of the power spectral density (PSD) in this band compared to the PSD in the overall signal 0 to 8 Hz provides a measure of the signal quality, which is defined as follows:^49^

$$\mathrm{rPSD}=\frac{\sum_{f=0.75}^{f=4}\mathrm{PSD}(f)}{\sum_{f=0}^{f=8}\mathrm{PSD}(f)},$$

where PSD was calculated using Welch’s method. The above can therefore be interpreted as the frequency-domain analog of PI.

***Skewness (skew)***: Skewness is a measure of the symmetry of a probability distribution. Krishnan et al. found that skewness is associated with corrupted PPG signals, which, intuitively, makes sense in the temporal domain.^50^ We also apply this metric to the frequency domain—from the Fourier transform. Intuitively, we assume a strong positive skew should be present in the spectral domain—when considering frequencies greater than 0.3 Hz—as a strong peak should be present between 0.75 and 3 Hz as a function of heart rate, whereas higher frequencies should be consistently lower in amplitude.

$$\text{skew}=\frac{\sum_{i=1}^{N}(x_{i}-\mu_{x}{)}^{3}}{(N-1)\sigma_{x}^{3}},$$

where $\mu_{x}$ and $\sigma_{x}$ are the empirical estimates of the mean and standard deviation of $x_{i}$, respectively, and N is the number of samples in the PPG signal. We computed this for both the time and frequency domains.

***Kurtosis (kurt)***: Recently, Selvaraj et al. found that kurtosis is a good indicator for PPG signal quality. Kurtosis is a statistical measure used to describe the distribution of observed data around the mean.^51^ It represents a heavy tail and peakedness, or a light tail and flatness, of a distribution relative to the normal distribution, which is defined as

$$\text{kurt}=\frac{\sum_{i=1}^{N}(x_{i}-\mu_{x}{)}^{4}}{(N-1)\sigma_{x}^{4}},$$

where $\mu_{x}$ and $\sigma_{x}$ are the empirical estimates of the mean and standard deviation of $x_{i}$, respectively, and N is the number of samples in the PPG signal. We computed this for both the time and frequency domains. In the case of good pulsatile data, we would expect there to be a strong peak in the pulsatile band in the frequency domain, while all other frequency bins—barring the sub-0.25 Hz region—should have relatively small magnitudes. In the case of poor pulsatile data, the distinction between the magnitude of the pulsatile and non-pulsatile regions—outside of the DC peak—is less, resulting in a more negative kurtosis.

***Autocorrelation ($\rho_{k}$)***: Autocorrelation is a measure of how much a signal correlates with itself across time; because PPGs exhibit a quasi-periodicity, we can leverage autocorrelation to measure the degree of agreement between the shape of peaks across time, thus giving an indication of signal quality. The equation is given by

$$\rho_{k}(t)=\frac{\text{sum}(y_{t}-\mu_{x})(y_{t-k}-\mu_{x})}{\text{sum}(y_{t}-\mu_{x})**2},$$

while the correlation between the red and NIR channel can also be used as a metric of degree of agreement between the two channels of absorption, it by itself does not provide information on the source of discrepancy—red or NIR channel. As such, the above method serves to better characterize each channel of absorption.

***Thresholds***: From empirical observation, we set thresholds for the exclusion of data analysis windows using the above metrics. Should an analysis window fail any single metric, the window is removed from consideration. Below are the thresholds for each metric:

*PI:* Less than $5\cdot 10^{-5}$

*rPSD*: Less than $5\cdot 10^{-9}$

*Spectral Kurtosis*: Less than 30

*Spectral Skew*: Less than 4

*Autocorrelation:* Less than 0.4

### 2.6 Experimental Design

To experimentally determine the impact of skin pigmentation, we made use of a live-porcine model developed by Drs. Mitch and Shmuylovich.^52^ The porcine model exhibits adjacent patches of pigmented and nonpigmented skin. We applied our devices to pigmented and nonpigmented patches of skin and deoxygenated the pig while recording light absorbance data from both devices. To accomplish this, we deoxygenated the pig from 100% ${\mathrm{SpO}}_{2}$, as measured from a separate pulse oximeter, to ∼70% ${\mathrm{SpO}}_{2}$ by modifying the ratio of oxygen and nitrogen gas inhaled. All ABGs were gathered from an indwelling arterial catheter and were run on an ABL 800 Flex. Approximately 10 ABGs were collected per deoxygenation cycle, whereas we simultaneously recorded red and NIR absorbance data from our custom pulse oximeters. All acquired data are then processed using our digital signal processing pipeline.

We repeated the above procedure on two pigs to obtain 16 full datasets; each pig had two pairs of devices placed on adjacent pigmented-nonpigmented sections, yielding four datasets per pig. Each pig also underwent two deoxygenation cycles, yielding eight datasets per pig. We then applied the same analysis pipeline described above to all 16 sets of data, extracted summary statistics, and ran an independent sample t-test to determine whether there was a statistical difference in the distribution of validation metrics between pigmented and nonpigmented skin. The second trial on the second pig had to be discounted due to poor device adhesion, resulting in failing to meet many validation metrics to extract modulation ratios from 88% to 100% ${\mathrm{SaO}}_{2}$, and so the final analysis was run on the remaining 12 datasets (six trials).

On the first pig, we acquired data from two deoxygenation cycles. In each deoxygenation cycle, we acquired data from two sets of pulse oximeters placed on the torso as shown in Fig. 3(d). On the second pig, we acquired data from one deoxygenation cycle, with one set of oximeters on the torso and one set on the ears of the pig. More specifically, because one ear was pigmented and the other nonpigmented, we placed one oximeter on each ear and compared the extracted data between the two ears.

### 2.7 Statistical Analysis

#### 2.7.1 Paired t-test (Fig. 4)

For each trial, we selected the time points we had ABG tests for and plotted the extracted metrics (R, AC, DC) for each time point. We then fit a linear regression line and found the slope and intercept. After repeating this procedure for all 12 datasets, we then ran a paired t-test comparing the slopes and intercepts between the pigmented and nonpigmented datasets and found the associated p-value. We set the degrees of freedom to n-3 to account for the number of parameters estimated—slope and intercept—in addition to the porcine model used.

**Fig. 4:**
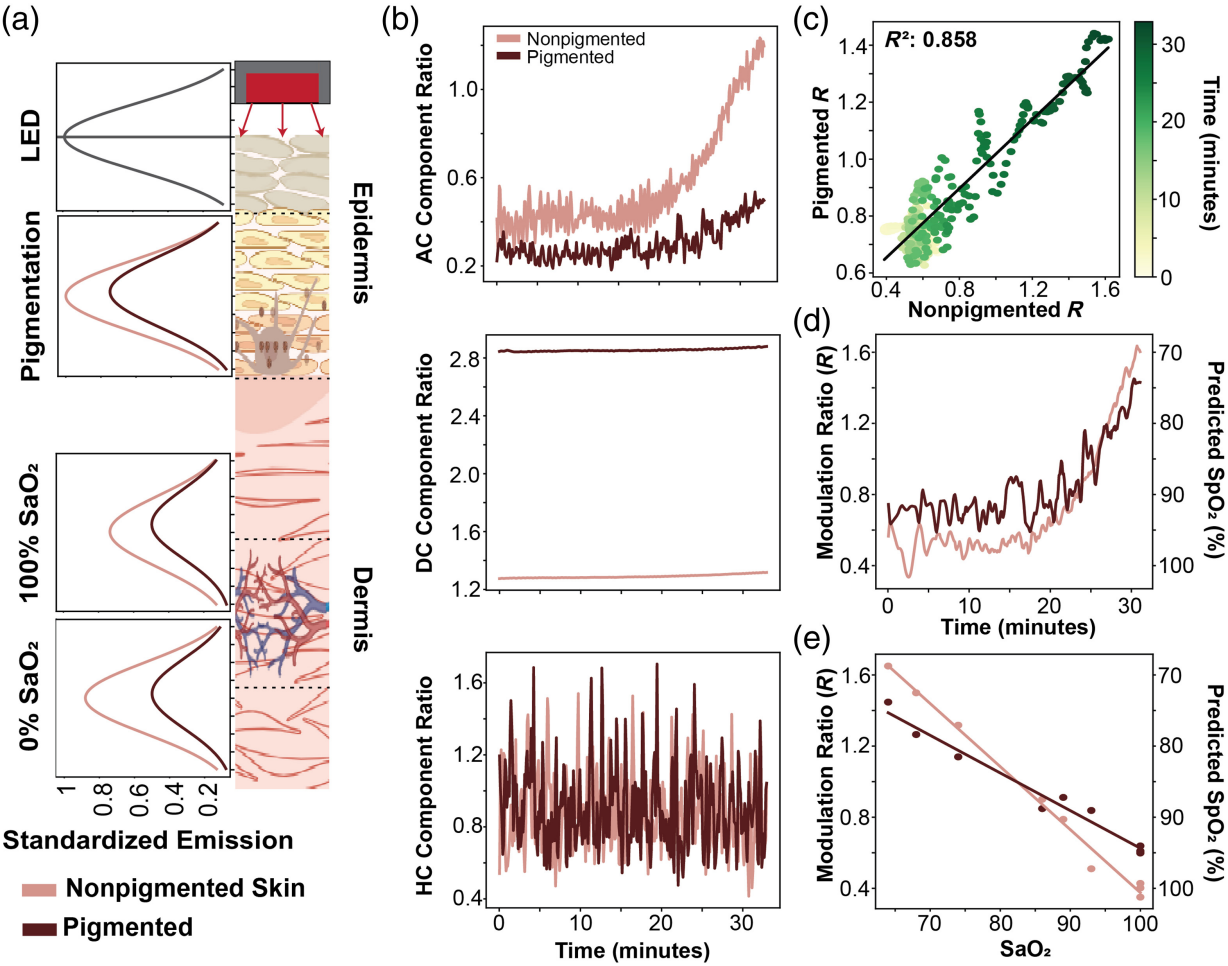
(a) Spectral emission of light from the LED (660 nm peak emission) prior to, and after, interaction with melanin and hemoglobin. (b) Baseline (DC), pulsatile (AC), and high-frequency (HC) component changes over time during a deoxygenation trial in pigmented and nonpigmented skin. (c) Correlation in extracted modulation ratio between pigmented and nonpigmented skin. (d) Modulation ratio across deoxygenation trial. (e) Extracted modulation ratios at each ABG test plotted with a line-of-best-fit.

#### 2.7.2 Regression t-test (Fig. 5)

We first calculated the difference between the nonpigmented and pigmented extracted metrics for each timepoint, across all relevant trials. Then, we selected the timepoints we had ABG tests for, thus providing a ground truth, and plotted a regression line. We then ran a t-test on the regression line parameters to determine if the slopes were truly non-zero, indicating there is a statistically significant difference in the change in each parameter across ${\mathrm{SaO}}_{2}$ levels. We set the degrees of freedom to n-3 to account for the number of parameters estimated—slope and intercept—in addition to the porcine model used.

**Fig. 5:**
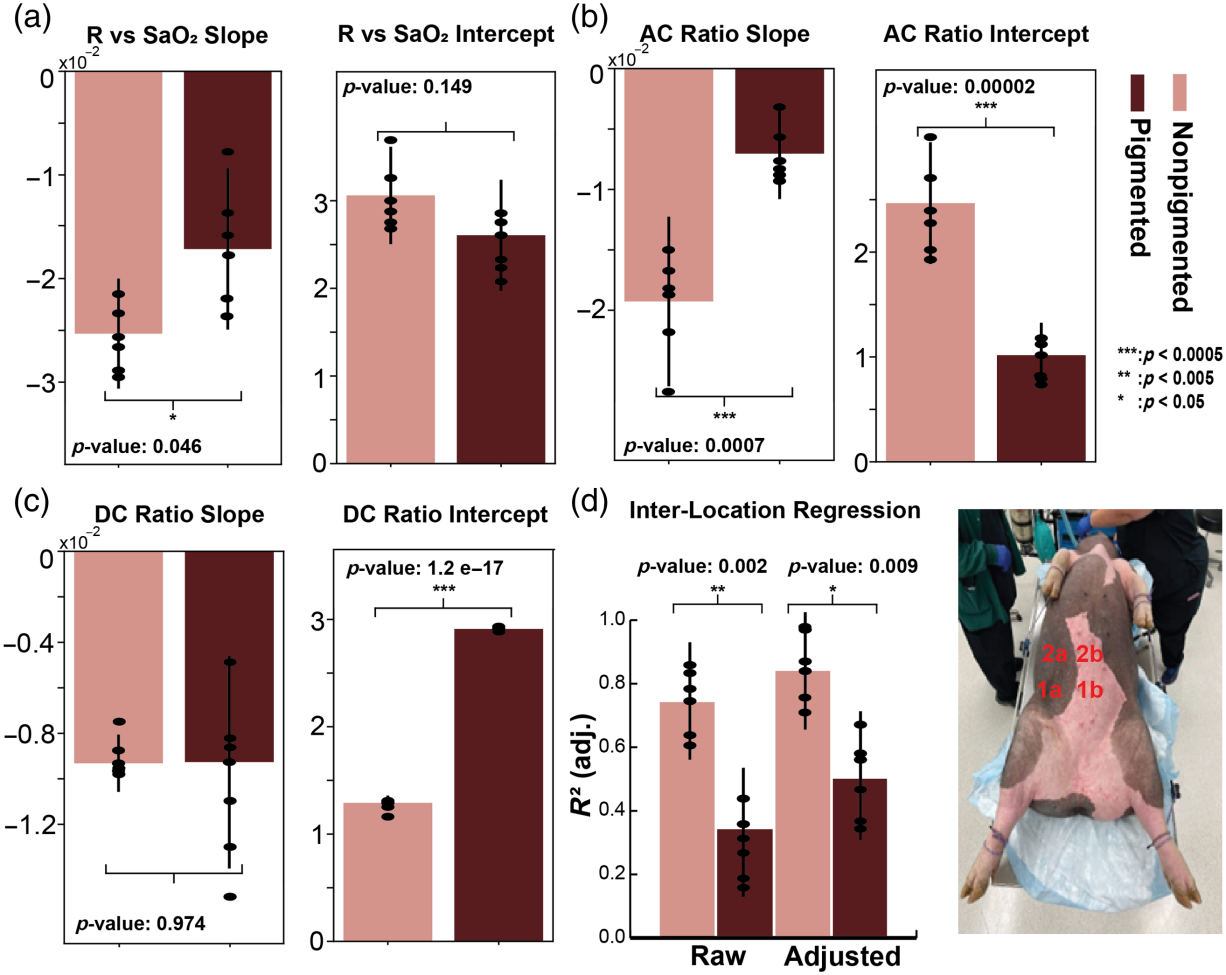
(a) Comparison of modulation ratio (R) versus ${\mathrm{SaO}}_{2}$ between skin pigmentations with associated t-test results. (b) Comparison of AC Ratio versus ${\mathrm{SaO}}_{2}$ between skin pigmentations with associated t-test results. (c) Comparison of DC Ratio versus ${\mathrm{SaO}}_{2}$ between skin pigmentations with associated t-test results. (d) Comparison of (adjusted) $R^{2}$ value between pigmented locations and nonpigmented locations with an associated diagram depicting where pairs of devices can be placed. Here, devices 1a and 1b provide pigmented and nonpigmented data for location 1, whereas devices 2a and 2b provide the same for location 2.

## 3 Results

Here, using the described live-porcine model, we demonstrate that the sensitivity of red light to changes in oxygen saturation is disproportionately attenuated in pigmented skin, thereby resulting in reduced sensitivity of pulse oximeters to changes in oxygen saturation in patients of color.

### 3.1 Experimental Characterization

To determine the impact of skin pigmentation on pulse oximetry, we made use of a live-porcine model where patches of skin exhibit darker skin pigmentation, and others lighter skin pigmentation. We then deoxygenated the pig from 100% ${\mathrm{SaO}}_{2}$, as measured from an ABG test, to ∼70% ${\mathrm{SaO}}_{2}$, by modifying the ratio of oxygen and nitrogen gas inhaled. ABGs were gathered from an indwelling arterial catheter and run on an ABL 800 Flex. For the duration of this trial, we simultaneously recorded data from two of our devices on a dark and light patch of skin. Each device continuously acquired the absorption of red and infrared light absorption across time. We then processed this data using our DSP pipeline and extracted pulsatile and baseline absorption ratios over time using both time and frequency domain metrics. Although acquiring data, several ABG tests were taken for each trial, allowing us to compare our extracted biometrics to a gold standard. Each trial was completed within 40 min.

Figure 4(b) matches our expectation regarding the extraction of AC, DC, and noise components. It is apparent that for the same reduction in oxygen saturation, the AC component ratio (red over infrared) changes dramatically less in pigmented skin. In addition, the DC component ratio (infrared over red) also appears to have a higher intercept in pigmented skin, suggesting the baseline measured absorbance of red light is significantly reduced in pigmented skin. Finally, the HC magnitude is equivalent between both pigmentations; however, because the absorption of red light is larger in pigmented skin, the red HC magnitude must be disproportionately higher for the HC magnitude to be similarly distributed regardless of pigmentation.

Figure 4(c) shows the correlation between the pigmented and nonpigmented data acquired in the same trial; if the two were identical, we would expect the $R^{2}$ value to be close to 1. Figures 4(d) and 4(e) show the extracted modulation ratio across time (c) and across ${\mathrm{SaO}}_{2}$ (d)—obtained from ABG tests acquired throughout the trial. The above shows the analysis pipeline outputs we rely upon for all six trials to determine the impact of skin pigmentation.

#### 3.1.1 Pigmented versus nonpigmented skin

Using the experimental setup described in the Experimental Design section, we then examine the linear regression lines fitted to each trial’s modulation ratio (R) versus ${\mathrm{SaO}}_{2}$, as shown in Fig. 4(e). More specifically, we examine the difference in slope and y-intercept of the regression lines calculated for each trial and compare by pigmentation type. In Fig. 5(a), we report a statistically significant difference in the slope of the regression lines between pigmented and nonpigmented skin, with pigmented skin reporting a lower slope magnitude. Looking at Fig. 5(b), we can observe the AC ratio is significantly different in slope and intercept between pigmented and nonpigmented skin, whereas Fig. 5(c) shows only the DC ratio y-intercept is significantly different between skin pigmentations, suggesting the observed difference in slope between the modulation ratio and ${\mathrm{SaO}}_{2}$ is largely a function of changes to the AC ratio.

In Fig. 5(d), we report the R-squared coefficients between multiple locations for each trial and pig in pigmented and nonpigmented sections of skin, as we depicted in Fig. 5(d). Here, we report a statistically significant difference in the R-squared values before and after adjustment (using our validation metrics to remove invalid points) between pigmented and nonpigmented skin, with pigmented skin having a lower R-squared value. This suggests regardless of whether validation metrics are applied, there is a statistically significant lower degree of agreement in ${\mathrm{SpO}}_{2}$ estimates in pigmented skin than in nonpigmented skin.

#### 3.1.2 Red versus NIR light

**Fig. 6:**
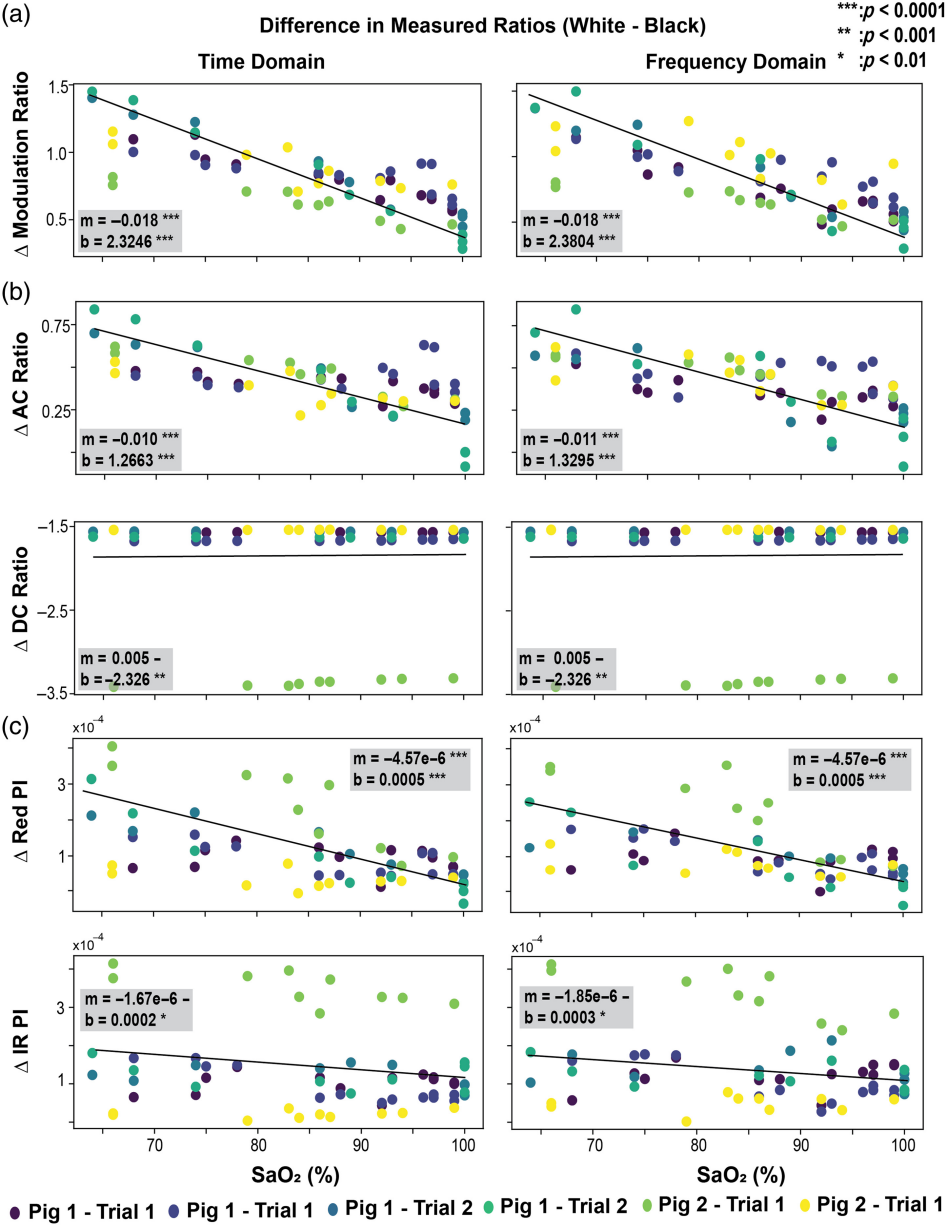
(a) Experimental difference of modulation ratio (R), (b) AC and DC ratio, and (c) red and infrared pulsatile index (PI) between pigmented and nonpigmented skin across six trials with associated regression t-test results.

In Figs. 6(a)–6(b), we extracted the modulation ratio (R), AC ratio, and DC ratio from each dataset using our PPG formulation from the time-domain and the frequency-domain. We then ran a t-test on the regression line parameters to determine if the slopes were truly non-zero, indicating there is a statistically significant difference in the change in each parameter across ${\mathrm{SaO}}_{2}$ levels. From these, we find that both the R and AC ratios are significantly different in their sensitivity (slope) across ${\mathrm{SaO}}_{2}$ levels. Critically, this difference is also observed in the frequency domain, suggesting the reduced sensitivity is not a function of poor temporal-domain signal quality. In addition, although we do see an intercept difference in the DC ratio, there does not appear to be a statistically significant difference in the sensitivity of the DC ratio to changes in ${\mathrm{SaO}}_{2}$.

The above confirms what we observe in Fig. 5; however, Fig. 6(c) shows how the pulsatile index (PI)—AC/DC for a given wavelength—changes for the red and NIR PPG data across ${\mathrm{SaO}}_{2}$. Here, it is apparent that the red channel exhibits reduced sensitivity in PI across ${\mathrm{SaO}}_{2}$ in pigmented skin, whereas the NIR channel is largely unaffected. This, combined with the observations in the AC and DC ratios, suggests the source of bias in the modulation ratio—and so the ${\mathrm{SpO}}_{2}$—comes from the disproportionate attenuation of the red pulsatile component in pigmented skin in hypoxia, thereby reducing the red pulsatile index, and in turn the sensitivity of pulse oximetry to decline in ${\mathrm{SaO}}_{2}$.

## 4 Conclusion

In this work, we presented a mathematical framework to describe PPG data in the time and frequency domains. Using this framework, we then characterized the impact of skin pigmentation on the extraction of baseline and pulsatile components in the red and near-infrared channels using both time and frequency-domain metrics. Here, we report a statistically significant reduction in the slope of the regression lines relating the modulation ratio and ${\mathrm{SaO}}_{2}$, as well as the AC ratio and ${\mathrm{SaO}}_{2}$, in pigmented skin. A reduction in sensitivity to changes in ${\mathrm{SaO}}_{2}$ would, in practice, result in an increasing overestimation of oxygen saturation in pigmented skin—a finding we observe in clinical settings as well. Bickler et al., for example, tested the extent of pulse oximeter oversaturation in black and white patients across a range of ${\mathrm{SaO}}_{2}$ levels, and found that pulse oximeter overestimation in black patients increases linearly as ${\mathrm{SaO}}_{2}$ decreases.^53^ In a systematic review of 44 studies, Martin et al. also found the extent of pulse oximeter overestimation in darker skin is greater at lower ${\mathrm{SaO}}_{2}$ than higher, further supporting findings from Bickler et al.^54^

From our mathematical framework, it is also apparent that skin pigmentation can impact the light–tissue interaction terms, including the attenuation coefficient and wavelength-dependent path-length terms. Because melanin absorbs—and melanosomes scatter—shorter wavelengths more than longer, we anticipate the red region would be more affected by skin pigmentation than the near-infrared region. From our experimental data, we also find the red pulsatile index—the ratio of the measured pulsatile absorption over baseline—is significantly more attenuated in pigmented skin, especially in hypoxia, whereas the NIR pulsatile index sensitivity is unaffected. This suggests that future work addressing pulse oximeter bias should focus on how light–tissue interactions in the red region are uniquely affected by skin pigmentation.

It should be noted that due to our limited testing on the impact of vascularization on pulse oximeter accuracy, we cannot confidently suggest that vascularization does not play a role in the observed pulse oximeter discrepancies across skin pigmentations. However, in our limited testing, we have found a greater degree of pulse oximeter agreement between core and peripheral (ear) locations in nonpigmented skin than in pigmented skin, suggesting skin tone likely plays a role in interpretability across tissues of various vascularization. For further discussion, please refer to Supplementary Material, Note 5. In addition, we also must acknowledge the limited sample size of our dataset and recognize the need for further testing to replicate these findings. Because we used only a reflectance-mode pulse oximeter in each experiment, we also acknowledge that further testing is required to confirm if our results are generalizable to transmission-mode pulse oximeters. Finally, because we kept LED brightness the same between devices placed on different skin pigmentation, it is possible that the poor signal quality in pigmented skin reduced the interpretability of our analysis. However, because we rely upon signal quality metrics to justify the inclusion of data for analysis, we believe the data points included for analysis can be reasonably interpreted.

Despite the limitations discussed above, the presented work provides insight into how skin pigmentation appears to attenuate pulse oximeter sensitivity at lower ${\mathrm{SaO}}_{2}$ levels, specifically through its impact on red-light absorbance. Importantly, the results from the experimental study also match existing clinical observations, thus furthering the applicability of our work to clinical contexts.

## Supplementary Material

10.1117/1.BIOS.2.3.032506.s01

## Data Availability

All raw data and associated code to reproduce the figures will be posted on our lab GitHub following publication of the paper, https://github.com/BailabUNC/
