# Supplementary material for: Characterizing the influence of skin pigmentation on pulse oximetry
Source: Biophotonics Discov. 2025 Jun 11;2(3):032506. doi: 10.1117/1.BIOS.2.3.032506 (PMC13052487; doi:10.1117/1.BIOS.2.3.032506)
Supplement: Supplementary file 1 [file BIOS_002_032506_SD001.pdf]

Supplementary Information

Supplementary Note 1: Tissue Morphology

Light first strikes the corneal layer of skin (stratum corneum), where 4-7% of light is reflected and the remainder passes through. Light then reaches the epidermis, the layer of skin where skin pigmentation arises from. Specifically, melanocytes - the cells responsible for the production of melanin in humans - reside in the basal layer of the epidermis where they form melanocyte-keratinocyte complexes. Depending on the location sampled, about 900-1500 melanocytes exist per square millimeter of skin[27]. When comparing the same body site, variation between individuals in melanocyte density is incredibly small even across different skin complexions[28 29]. However, in the cytoplasm of melanocytes exist special membrane-bound organelles called melanosomes. It has been found highly pigmented skin can contain up to five times more melanosomes than lightly pigmented skin[30]. Additionally, these melanosomes can be larger, dispersed among the surrounding keratinocytes (as opposed to being membrane-bound clusters), and produce larger quantities of melanin - the specific pigment responsible for absorbing certain wavelengths of light. Jacques et al. reported the volume fraction of melanin in the epidermis is 1-3% for light-skinned Caucasians, 11-16% for Mediterranean, and 18-43% for Africans[31 32]. According to Chatterjee et al. the majority of red light is absorbed within the epidermis, while a significant portion of infrared light is absorbed by both the epidermis and dermis[35].

Once in the dermis, light undergoes Rayleigh scattering due to the collagen fibrils in the papillary dermis. Light that enters the reticular dermis then undergoes Mie scattering due to the larger collagen fibers and is preferentially directed deeper to the subcutis. A proportion of light will be absorbed by hemoglobin within the blood in the capillary networks in the dermis as well[34]. It is this blood, especially in the upper capillary network, that we are relying upon to assess oxygenation.

While there are many other chromophores to consider, for the purposes of our discussion on skin pigmentation, the above are sufficient to understand the context of pulse oximetry.

Impact of Melanin Sub-Types

While we have thus far referred to melanin as a singular unit, there are multiple types of melanin present in human skin, most significant of them being the black-brown eumelanin and red-yellow pheomelanin[55]. Additionally, there are further subdivisions to the aforementioned types of melanin; for example, eumelanin may be classified as DHI-rich, characterized by darker brown or black, or DHICA-rich, characterized by a lighter brown color[56]. As such, it is important to note that higher proportions of pheomelanin could yield higher km values, while higher proportions of DHI-rich eumelanin could lower the km value. The consequence of this is that for the same incident wavelength, DHI-rich eumelanin will absorb more light than pheomelanin will; more generally, the presence of melanin - regardless of type - will contribute to the net absorbance of light passing through biological tissue. In terms of human populations, the above statements translate to two features: individuals producing melanin, regardless of type, will likely absorb larger proportions of incident light, and individuals with a greater proportion of eumelanin - and so more brown or black skin - will absorb more incident light than those with greater proportions of pheomelanin.

Supplementary Note 2: Light-Scattering in Melanin

Generally, scattering in the skin can be described by the following equation:

$$\mu_s'(\lambda) = \mu_s'(\lambda_s)(\lambda/\lambda_s)^{-\gamma},$$

where λ_s is 1000 nanometers, and γ is the scattering power.

While no theoretical model has established its general validity, several proposed biological tissue scattering models - from Mie theory models to Born approximation models - predict the power law dependence described[57 58 59]. The exponent, γ, refers to the scattering power of tissue where smaller values indicate larger scatterer sizes in tissue and larger values indicate smaller scatterer sizes. The value for gamma when defining the scattering coefficient for skin could therefore be understood as the weighted average scatterer size in skin. Zonios et al. presented a brief literature review where they found a wide range of gamma values, and suggested that some of this variation may be due to differences in measurement techniques. Additionally, while Rajadhakshya et al. found that scattering due to melanin provides strong contrast in the skin when using confocal microscopy[60], Zonios et al. found no significant difference in light scattering between different skin pigmentations when using diffuse reflectance spectroscopy[61]. This observation suggests melanin affects primarily the absorption of light, not scattering.

Supplementary Note 3: Instrumentation

Light-Emitting Diode

Light Emitting Diodes (LEDs) serve as a non-thermal source of light radiation in portable spectroscopy, relying on the recombination of electrons and electron-holes to produce light. The wavelength, and in turn the energy, of the emitted light depends on the energy band gap of the semiconductor used.

LEDs are relatively sensitive to ambient temperature in terms of light intensity and peak spectral emission. Generally, an increase in temperature result in relative intensity reduction and a shift to longer wavelengths (red-shifts) in peak emission. From experimental results, the relation between LED emission intensity (I) and temperature (T) is often given as: $I = I_{300K}e^{(T-300)/(-T_1)}$, where T_1 is the characteristic temperature for a given LED[63] Theoretically, the wavelength (λ) of emitted light is given by $\lambda \approx \frac{hc}{E_{bg}}$, where h is Plank's constant, c the velocity of light, and E_{bg} the bandgap energy. The bandgap energy can in turn be expressed as a function of temperature (T): $E_{bg}(T) = E_{g0} - \frac{\alpha T^2}{T+\beta}$ where E_{g0} is the bandgap energy when T=0 kelvin, and α & β are fitting parameters depending on the material. The above suggests temperatures above room temperature reduce emitted light intensity, and shift peak spectral emission to longer wavelengths.

Additionally, while modern LEDs utilize quantum wells to improve wavelength emission specificity, there is still a significant variance in spectral emission. This implies the recorded absorption is not a point measurement at a given wavelength, but instead a convolution of the LED emissivity curve with respect to the target chromophore absorptivity.

Photodiode

Photodiodes are semiconductor devices that produce electrical current in response to photon absorption. Fundamentally, photodiodes are PN junction diodes, formed by doping a section of a x-type bulk silicon wafer, with an anti-reflection coating applied over the doped (active) area of the diode, reducing the reflection of the target wavelength. It should be noted, while the anti-reflection coatings due improve the selectivity of photodiodes to incident wavelengths, they still accept a broader range of wavelengths than required for pulse oximetry. As such, the output of a photodiode is best understood as a convolution of the photodiode relative absorption curve and incident light intensity across all wavelengths.

There are three properties of photodiodes that contribute to its signal quality: series resistance, junction capacitance, and shunt resistance. Both series resistance and junction capacitance arise from, and are largely static, structural properties of the semiconductor (such as substrate width and

resistivity). However, shunt resistance – the resistance of a zero-biased photodiode junction – arises from material impurities and is inversely related to Johnson (thermal) noise (I_{jn}), defined as $I_{jn} = \sqrt{\frac{4k_B T\Delta f}{R_{sh}}}$, where k_B is the Boltzmann Constant, T is temperature, and Δf the noise measurement bandwidth. From this, we know an increase in temperature or reduction in shunt resistance increases the thermal noise of the photodiode.

Impact of Instrumentation

As mentioned earlier, in response to increase in ambient temperature LEDs will experience a shift in peak spectral emission to longer wavelengths, and a relative reduction in emitted light intensity. According to Bozkurt and Onaral, while NIR LEDs can elevate skin temperature to upwards of 10 degrees Celsius, majority of this is due to conducted heat from the semiconductor junction, with only a 0.5 degree change being attributed to NIR absorption[64]. Additionally, Grandinetti et al. found no significant increase in skin temperature across different skin color groups when concurrently using pulsed red and infrared LEDs and super-pulsed lasers at upwards of 50 J[64]. It should be noted, however, that the threshold for red light irradiation before adverse events may be lower in skin of color (320 J/cm^2) than otherwise (480 J/cm^2), suggesting pigmented skin may still be more effected by irradiation[65].

Similar to LEDs, photodiodes also exhibit a temperature-dependence in output current. Dark current – the leakage current that flows when a bias voltage is applied without any accompanying light – appears directly correlated with temperature. Specifically, dark current approximately doubles with every 10-degree increase in temperature, while shunt resistance doubles for every 6-degree increase 67. To our knowledge, the change in dark current output appears unrelated to the target wavelength. Additionally, within the context of pulse oximetry, we are unsure if there is a significant difference in temperature between patients of varying skin pigmentation. If there were, we would expect the Johnson noise from the photodiode to be larger in pigmented skin than in non-pigmented skin. However, because absorption and scattering of red and NIR light by melanin is relatively high, the contribution of temperature differences to photodiode noise is likely to be relatively insignificant.

From the above, it is difficult to predict any consistent trend in instrumentation signal quality due to skin pigmentation. In the event pigmented skin increases in temperature significantly more in response to similar irradiation, we would expect the following changes in pigmented skin: a shift in peak emitted wavelength to slightly longer wavelengths, a reduction in relative emitted light intensity, and an increase in photodiode noise due to larger dark current intensities. Given the evidence for substantial differences in skin temperature across pigmentation levels is minimal, we cannot ascribe clinical findings solely to the instrumentation of pulse oximeters.
However, the relative absorption curves of both the LED and photodiode are likely to play a significant role in the quality of the acquired data across a range of SaO_2 levels due to the predicted rightward shift in relative peak transmission wavelength particularly in the red region. Should the relative emissivity of an LED allow for a broader range of emitted wavelengths, the predicted peak shift would likely be more dramatically different between skin tones at lower SaO_2 levels as well. Additionally, the photodiode relative absorption curve can, if selected appropriately, attenuate extraneous wavelengths of light, thereby mitigating the impact of the predicted peak-wavelength shift.

Supplementary Note 4: Fourier Transform

In practice, a Fast Fourier Transform (FFT) is often used to extract biometrics from the input PPG signal. The FFT is a variant of a Discrete Fourier Transform (DFT), a method that allows an input time-series signal can be represented in the frequency domain through the following transformation:

$$F_k = \sum_{n=0}^{N-1} f_n e^{-2\pi ikn/N} \ for\ 0 \leq k \leq N-1$$

where N is the number of time points, and frequency is represented by $\frac{k}{N}$. Since Euler's formula gives us $e^{2\pi ikn/N} = \cos\left(\frac{2\pi kn}{N}\right) + i \cdot sin\left(\frac{2\pi kn}{N}\right)$, the computation of F_k effectively gives us the coefficients of an approximation of the signal by a linear combination of the real and imaginary components.

To extract the pulsatile and baseline components of the input signal, it is possible to apply the DFT to the input signal, then compute the inverse Discrete Fourier Transform (iDFT) for a target frequency region, thus removing the influence of extraneous frequencies. The iDFT is given by the following transformation:

$$f_n = \frac{1}{N} \sum_{k=f_1}^{f_2} F_k e^{2\pi ikn/N} \ for\ f_1 \geq 0\ \&\ f_2 \leq N-1$$

where f_1 and f_2 are the bounds for the target frequency region. FFTs are also useful for characterizing the quality of an input signal, as we discuss in our *Signal Quality Metrics.*

Supplementary Note 5: Core vs Peripheral Pulse Oximeter Differences

Because peripheral and core vasculature may respond differently to deoxygenation, we placed devices on the ears and torso of the porcine model, and recorded data from all four pulse oximeters simultaneously to determine the impact of location and vascularity on pulse oximeter discrepancy. From ***SI Fig. 1***, it is apparent the data between the ear and torso in nonpigmented skin is much more correlated than in pigmented skin, with the R^2 value being 0.933 in nonpigmented skin and 0.620 in pigmented skin. While the sample size is too small to generalize these results, it does suggest peripheral and core locations by themselves aren't necessarily the key sources of discrepancy, as nonpigmented skin in this porcine trial perform consistently across region. To confirm this, further testing is required.

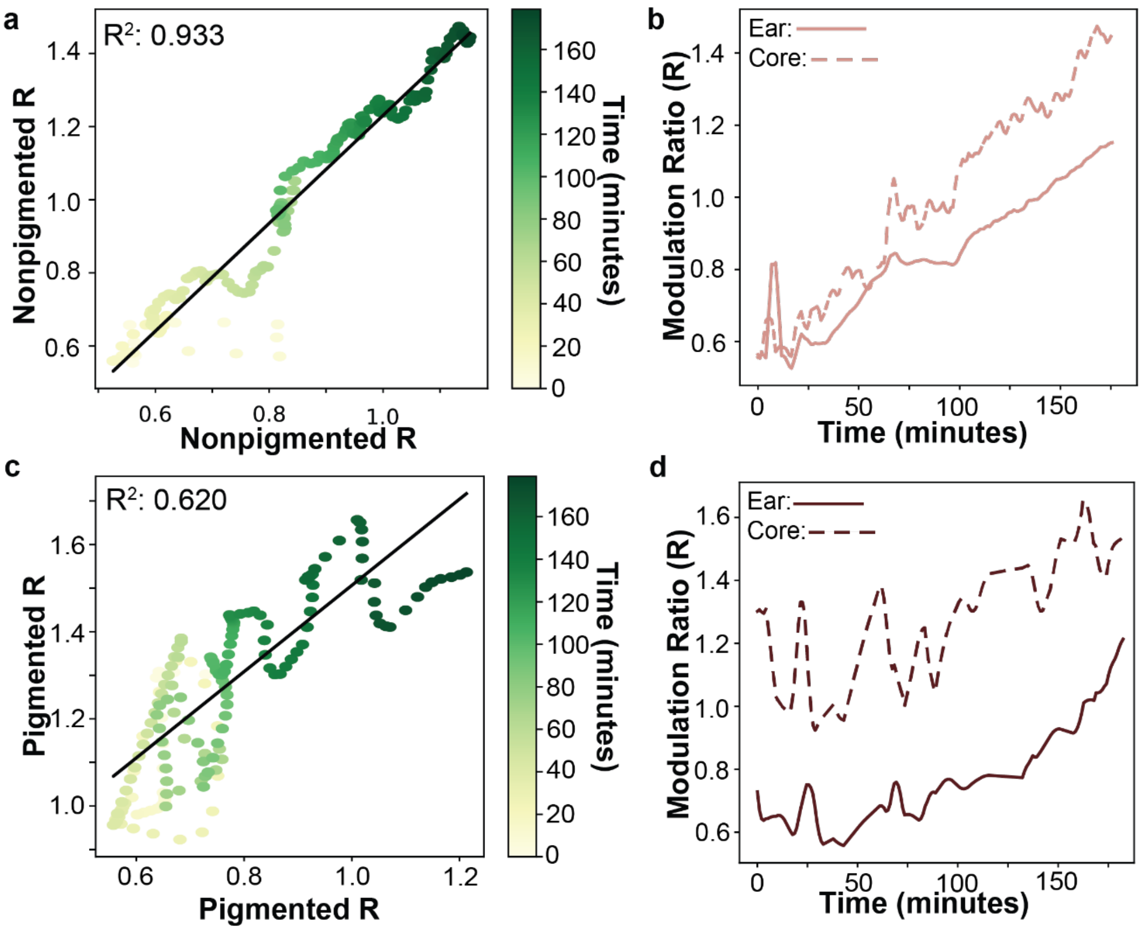

Supplementary Figure 1: (a) Correlation between devices placed on nonpigmented ear and core (b) Modulation ratio vs time for devices in a (c) correlation between devices placed on pigmented ear and core (d) Modulation ratio vs time for devices mentioned in c.
